# Factors affecting parental decisions for termination of pregnancy in the presence of a prenatal trisomy 21 diagnosis

**DOI:** 10.1590/1806-9282.20250193

**Published:** 2025-10-27

**Authors:** Ayça Peker, Atakan Tanaçan, Nurşen Özen, Göksun İpek, Gülcan Okutucu, Zahid Ağaoğlu, Esra Şükran Çakar, Dilek Şahin

**Affiliations:** 1 Turkish Ministry of Health, Ankara Bilkent City Hospital, Department of Obstetrics and Gynecology, Division of Perinatology – Ankara, Turkey.; 2 Ministry of Health, Ankara Bilkent City Hospital, Department of Obstetrics and Gynecology – Ankara, Turkey.; 3 Ministry of Health, Ankara Bilkent City Hospital, Department of Medical Genetics – Ankara, Turkey.; 4 Turkish Ministry of Health, University of Health Sciences, Ankara Bilkent City Hospital, Department of Obstetrics and Gynecology, Division of Perinatology – Ankara, Turkey.

**Keywords:** Fetal anomalies, Down syndrome, Abortion, induced, Chromosome aberrations, Prenatal diagnosis

## Abstract

**OBJECTIVE:**

The aim of the study was to research the effective factors on decision-making process to terminate or continue the pregnancy in trisomy 21.

**METHODS:**

A total of 103 patients who had a confirmed trisomy 21 diagnosis in a tertiary center were involved in this study. Demographic data, obstetric history, educational status, family income, and maternal employment were compared between termination of pregnancy and continuation of pregnancy (non-termination of pregnancy) groups. Also, possible effective factors on parents’ decisions were evaluated by a questionnaire.

**RESULTS:**

Maternal age, number of miscarriages, number of living children, maternal and paternal educational status, family income, maternal working status, and detected fetal anomalies were not found to be effective on the decision-making process. Early diagnosis was correlated with a higher termination of pregnancy ratio. Psychological and economic factors, concerns of having less time and energy for other children, and career concerns were significantly effective in the termination of pregnancy group. While in the non-termination of pregnancy group, religious factors were the main concern.

**CONCLUSION:**

Gestational age at diagnosis, psychological factors, economic status, and religious concerns seem to be the possible factors behind deciding termination of pregnancy in pregnancies with trisomy 21.

## INTRODUCTION

Trisomy 21, also called Down Syndrome (DS), is the most common chromosomal abnormality in infants. Its frequency is nearly 1/800 births worldwide. Also, it is the most frequent chromosomal abnormality associated with intellectual disability. Intellectual disability in DS is mostly moderate, but it can change from mild to severe^1^.

Structural birth defects are more prevalent in individuals with DS compared to the general population. Congenital cardiac anomalies are the most frequent, occurring in about 44–58% of newborns with DS^1–3^. Additionally, the gastrointestinal, orofacial, musculoskeletal, and nervous systems can also be affected^3–6^. These conditions contribute to lifelong morbidity and increased mortality among affected individuals, underlining the importance of prenatal diagnosis for trisomy 21.

The primary goal of prenatal diagnosis is to inform families and allow them the option to decide whether to continue or terminate the pregnancy. This decision-making process can be challenging, particularly when the pregnancy was planned and desired.

The present study explores the factors influencing the decision-making process for the termination of pregnancies (TOPs) affected by trisomy 21. This study also aims to shed light on all aspects such as, the psychological, economic, and religious factors and other concerns, comparing TOP and non-TOP (continuation of pregnancy) groups.

## METHODS

This retrospective descriptive study was conducted at the Perinatology Clinics of Ankara Bilkent City Hospital, a tertiary referral center with a high influx of patients from across the country. Conventional prenatal diagnostic tests, such as chorionic villus sampling (CVS) or amniocentesis (A/S) and cordocentesis, were performed for this high-risk patient group. The study included patients whose invasive diagnostic tests confirmed trisomy 21 between October 2019 and March 2024.

Indications for invasive testing included increased risk based on screening test results, the presence of soft markers, positive noninvasive prenatal test (NIPT) results, or parental request. The choice between CVS and amniocentesis depended on the gestational age at presentation, risk factors, specific indications, patient preference, and clinician discretion.

All pregnant women diagnosed with trisomy 21 through invasive diagnostic testing are counseled by a maternal-fetal medicine specialist in our hospital. The specialist explains all possible complications associated with trisomy 21, informs the family of their legal right to terminate the pregnancy if they wish, and discusses available termination methods along with their potential risks and complications. In addition, all families are consulted with specialists from the social pediatrics, neonatology, psychology departments, and social services. During these consultations, sufficient time is dedicated to providing detailed information to each family, consistent, standardized information is provided, and all questions from the families are carefully addressed. Following these discussions, families are supported in making their decision regarding the pregnancy. In both cases, they choose to terminate or continue the pregnancy; an informed consent form is obtained from all families.

Maternal age and obstetric history were documented. Data gathered from ultrasonography, including major fetal anomalies, cardiac anomalies, or soft markers for aneuploidies, were recorded. Sonographic findings such as cystic hygroma, hydrops, atrioventricular septal defect (AVSD), ventricular septal defect (VSD), hydrocephalus, double bubble sign, and diaphragmatic hernia were classified as major fetal abnormalities. Hospital records were utilized to obtain the indications for invasive prenatal tests, sonographic findings of fetuses, and the gestational age at the time of karyotyping and final diagnosis.

Retrospectively, a questionnaire was administered to patients who consented to participate in the study. Both the TOP group and the non-TOP group were queried regarding the factors influencing their decision-making process. The interviews covered aspects such as the educational status of both parents, family income, and factors influencing their decisions, including psychological, economic, and religious considerations; career plans; concerns about having less time and energy for other children; the influence of grandparents; and the impact of medical professionals.

Prior to conducting the study, we reviewed the existing literature on decision-making processes regarding pregnancy termination and analyzed the questionnaires previously used. Taking into account Turkey's secular structure and predominantly Muslim population, we developed a unique, culturally appropriate questionnaire tailored to our study population.

The content validity of the questionnaire was assessed following the methodology outlined by Lawshe^7^. Nine experts with specialization in maternal-fetal medicine evaluated each item, classifying them as: (1) Essential, (2) useful but not essential, or (3) not necessary. The content validity ratio (CVR) for each item was computed based on the degree of consensus among the experts. Referring to Lawshe's critical value table, which recommends a minimum CVR of 0.78, all items met the required threshold and were considered essential^7^. As a result, the overall content validity index (CVI) was calculated as 1.00, signifying unanimous expert agreement regarding the questionnaire's relevance and alignment with its intended objectives. To further evaluate item clarity and response behavior, a pilot test was conducted with five individuals from the target population. Based on the feedback received, minor modifications were made, leading to the finalization of the questionnaire.

The study was conducted in accordance with the Declaration of Helsinki and received approval from the Institutional Review Board of the University of Health Sciences, Turkey, Ankara Bilkent City Hospital Ethics Committee (approval number: E2-24-6141).

Statistical analyses were conducted using Statistical Package for the Social Sciences (SPSS) version 23 (IBM, Armonk, NY, USA). Descriptive statistics were reported as median and interquartile range (IQR), as the data did not follow a normal distribution. The Mann-Whitney U test was used to compare parameters between groups. Categorical variables were expressed as counts and percentages, with the χ² test employed for comparing these variables between groups. A multivariate logistic regression was used to derive factors and their relative weight on the decision-making process of parents. A two-sided p-value of less than 0.05 was considered statistically significant.

## RESULTS

A total of 118 patients were diagnosed with trisomy 21 through prenatal testing between October 2019 and March 2024 in Ankara Bilkent City Hospital's Perinatology Department. We were able to reach 98 patients, who provided informed consent to participate in this study. 68 patients opted for pregnancy termination, while 30 chose to continue their pregnancies. One patient with a dichorionic-diamniotic twin pregnancy elected to undergo a feticide procedure for the fetus with trisomy 21, while preserving the healthy twin; thus, this patient was included in the termination group.

Within the non-TOP group, one patient experienced intrauterine fetal death at the 27th week of gestation. Two patients had preterm delivery and lost their babies after birth. Seven patients developed fetal growth restriction during follow-up.

When evaluating demographic data, no significant differences were observed between TOP and non-TOP groups (Table 1). The overall median maternal age was 35, and it was similar between the two groups. Variables such as maternal age, gravidity, parity, history of miscarriages, and the number of living children were comparable. Additionally, the educational status of the mother and father, the monthly family income, and the maternal employment status were similar between the two groups.

**Table 1:** Demographic data of termination of pregnancy and non-termination of pregnancy groups.

		TOP group	Non TOP group	p-value
Maternal age (median, IQR)		35 (10)	34.5 (11)	0.87
Gravidity (median, IQR)		3 (1)	3 (3)	0.05
Parity (median, IQR)		1 (2)	2 (1)	0.07
Miscarriage (median, IQR)		0 (1)	0 (2)	0.32
Live children (median, IQR)		1 (2)	2 (1)	0.09
Maternal educational status (n, %)				
	Elementary	5 (7.4)	3 (10)	
	Lower secondary	13 (19.1)	4 (13.3)	
	Upper secondary	28 (41.2)	13 (43.3)	0.98
	Bachelor's degree	20 (39.4)	9 (30)	
	Master's/Doctorate degree	2 (2.9)	1 (3.3)	
Paternal educational status (n, %)				
	Elementary	6 (8.8)	2 (6.7)	
	Lower secondary	16 (23.5)	7 (23.3)	
	Upper secondary	22 (32.4)	11 (36.7)	0.94
	Bachelor's degree	21 (30.9)	8 (26.7)	
	Master's/Doctorate degree	3 (4.4)	2 (6.7)	
Family income (n, %)				
	Low income	29 (42.6)	6 (20)	
	Middle income	37 (54.4)	24 (80)	0.14
	High income	2 (2.9)	0 (0)	
Maternal working status (n, %)				
	Working mom	22 (32.4)	8 (26.7)	0.69
	Non-working mom	46 (67.6)	22 (73.3)	

TOP: termination of pregnancy; IQR: interquartile range.

The most common indications for invasive diagnostic tests were increased risks in prenatal screening tests, cystic hygroma and increased nuchal translucency measurement. Thirty-five patients underwent CVS, while 67 had amniocentesis (A/S). In the TOP group, 28 patients (42%) had CVS, and 40 patients (58%) had A/S. In non-TOP group, five patients (17%) had CVS, 24 patients (80%) had A/S, and one patient (3%) had cordocentesis. This patient presented at 30 weeks of pregnancy with a double bubble sign and underwent a cordocentesis process for karyotyping.

The median karyotyping week was 13 weeks in the TOP group and 15 weeks in the non-TOP group. The median gestational age at diagnosis was 16 weeks for the TOP group and 17,5 weeks for the non-TOP group. The gestational age at the time of trisomy 21 diagnosis had a significant influence on the parents’ decisions. Statistically, families with an earlier diagnosis exhibited a higher termination rate (p<0.01).

Among the patients, four had conceived through in vitro fertilization (IVF); three of these chose to terminate their pregnancies, while one experienced a miscarriage at 17 weeks.

No statistically significant differences were observed between the groups in terms of soft markers, fetal cardiac anomalies, or other major anomalies (Table 2). The most frequently observed soft markers were nasal bone hypoplasia, increased nuchal translucency, and increased nuchal fold thickness. Among major cardiac anomalies, AVSD was the most prevalent. Other major anomalies observed in fetuses with trisomy 21 included cystic hygroma, duodenal atresia, tetralogy of Fallot, hydrocephalus, omphalocele, diaphragmatic hernia, VSD, acrania, cleft lip, and hydrops.

**Table 2:** Factors affecting termination of pregnancy decision.

		TOP group	Non TOP group	p-value
	Gestation week at diagnosis (median, IQR)	16 (4.8)	17.5 (4)	<0.01
Major fetal anomaly (n, %)				
	Yes	29 (42.6)	12 (40)	0.24
	No	39 (57.4)	18 (60)	
Soft markers (n, %)				
	Yes	42 (61.8)	17 (56.7)	0.6
	No	26 (38.2)	13 (43.3)	
Major fetal cardiac anomaly (n, %)				
	Yes	16 (23.5)	10 (33.3)	0.57
	No	52 (76.5)	20 (66.7)	
Were psychological factors effective on your decision? (n, %)				
	Yes	25 (36.8)	2 (6.7)	<0.01
	No	24 (35.3)	22 (73.3)	
	Not sure	19 (27.9)	6 (20)	
Were economic factors effective on your decision? (n, %)				
	Yes	18 (26.5)	2 (6.7)	0.01
	No	33 (48.5)	26 (86.7)	
	Not sure	17 (25)	2 (6.7)	
Were religious factors effective on your decision? (n, %)				
	Yes	3 (4.4)	23 (76.7)	<0.01
	No	61 (89.7)	3 (10)	
	Not sure	4 (5.9)	4 (13.3)	
Was the concern of having less time and energy for your other children effective on your decision? (n, %)				
	Yes	20 (29.4)	2 (6.7)	<0.01
	No	23 (33.8)	25 (83.3)	
	Not sure	25 (36.8)	3 (10)	
Were your career concerns effective on your decision? (n, %)				
	Yes	8 (11.8)	0 (0)	<0.01
	No	54 (79.4)	28 (93.3)	
	Not sure	6 (8.8)	2 (6.7)	
Were your own parents effective on your decision? (n, %)				
	Yes	3 (4.4)	4 (13.3)	0.61
	No	60 (88.2)	24 (80)	
	Not sure	5 (7.4)	2 (6.7)	
Were medical professionals effective on your decision? (n, %)				
	Yes	13 (19.1)	2 (6.7)	0.03
	No	45 (66.2)	26 (86.7)	
	Not sure	10 (14.7)	2 (6.7)	

TOP: termination of pregnancy; IQR: interquartile range.

In interviews with the patients, factors influencing the decision-making process were examined (Table 2). Psychological and economic considerations were significantly more influential in the TOP group than the non-TOP group (p=0.003 and p=0.01, respectively). All the participants identified themselves as Muslim. However, religious considerations were more influential in the non-TOP group (p<0.01). Between the two groups, concerns regarding the allocation of time and energy to other children, as well as career considerations, were more significant in the TOP group (both p<0.01). The influence of grandparents was not a significant factor in the decision-making process for either group. However, medical professionals were found to have a significantly greater impact on the decision-making process in the TOP group (p=0.03). As shown in Table 3, multivariate regression analysis indicated that gestational age at diagnosis, psychological, economic, and religious factors significantly influenced parental decision-making.

**Table 3:** Multivariate regression analysis of factors affecting termination of pregnancy decision.

	p-value	OR	95%CI
Gestation week at diagnosis	0.05	0.679	0.459–1.005
Were psychological factors effective on your decision?	0.139	4.522	0.613–33.366
Were economic factors effective on your decision?	0.05	6.183	0.930–41.115
Were religious factors effective on your decision?	<0.001	0.83	0.027–0.262
Was the concern of having less time and energy for your other children effective on your decision?	0.06	6.719	0.924–48.865
Were your career concerns effective on your decision?	0.987	1.041	0.008–143.263
Were medical professionals effective on your decision?	0.197	2.909	0.574–14.740

OR: odds ratio; CI: confidence interval.

## DISCUSSION

Prenatal diagnosis has critical importance for trisomy 21. After a diagnosis of DS, parents in many countries have the legal right to terminate the pregnancy. The American College of Obstetricians and Gynecologists (ACOG) recommends offering prenatal screening and diagnostic tests to all patients, emphasizing the importance of adequate counseling to discuss the benefits and limitations of these tests^8^.

In Turkey, prenatal screening for DS is widely conducted in the first and/or second trimester, following the guidelines of the Turkish Ministry of Health. When screening test results indicate elevated risk, patients are referred to tertiary centers for further evaluation and to conventional invasive diagnostic procedures.

The legal limit for abortion in Turkey is 10 weeks of gestation. But there are no limitations for TOP if DS or major fetal anomalies are present or for cases that threaten the mother's health and life. So, after a confirmed trisomy 21 diagnosis by invasive diagnostic tests, pregnancy can be terminated with the family's demand and approval of three physicians.

This study analyzed factors influencing the decision to terminate or continue pregnancies complicated by DS. The median maternal age at diagnosis was 35 years, consistent with literature indicating that advanced maternal age is a risk factor for aneuploidies^9,10^. Maternal age, number of pregnancies, and live children were thought to be effective on patients’ decisions. But there was no statistically significant influence of these factors. However, previous studies have reported a relationship between advanced maternal age and increased rates of pregnancy termination^11,12^. Among the participants, four had conceived via IVF, and three of these chose to terminate the pregnancy, suggesting no significant tendency against termination following assisted reproductive technologies (ART).

The gestational age at diagnosis was earlier in the termination group, suggesting that earlier diagnoses may influence the decision to terminate pregnancies affected by trisomy 21.

In the univariate analysis, psychological and economic factors, concerns regarding career, limited time and energy for other children, and the influence of medical professionals were found to affect the decisions of the TOP group. However, in the multivariate analysis, only psychological and economic factors remained significant. Additionally, the multivariate analysis revealed that religious factors played a significant role in the decision-making of the non-TOP group.

In a retrospective study, an inverse correlation was identified between the gestational age at the time of karyotyping and diagnosis, and the decision to terminate pregnancies affected by trisomy 21. Consistent with our findings, maternal age, gravidity, parity, a history of early pregnancy loss, and the presence of major fetal anomalies were not found to have a significant impact on parental decisions regarding pregnancy termination^13^.

Maternal and paternal educational status, family income, and the working status of the mother might be important for the decision. A child with trisomy 21 has not only intellectual disability, may also have many other medical problems as mentioned before. That could be challenging for the family in terms of money and time. However, educational status, family income, and working status of the mother were not significantly different between TOP and non-TOP groups.

The presence of major fetal anomalies is often considered a valid reason for pregnancy termination, particularly when these anomalies are life-threatening or severely disabling. However, this study did not find a significant relationship between major fetal anomalies and termination rates, aligning with findings from other studies^14,15^.

A long-term prospective study by Schechtman et al. analyzed over 53,000 pregnancies and identified correlations between maternal age and educational status with termination rates when major fetal anomalies were present^16^.

A recent study by Hawkins et al declared that there was no significant correlation between maternal age, gestational age, fetal abnormalities, and termination rates^17^.

A previous study from Turkey did not find a significant relationship between maternal age, obstetric history, early diagnosis, or the presence of major fetal anomalies and termination decisions in trisomy 21 cases, though it reported that higher education, maternal employment, and family income were associated with increased termination rates. In this study, all 12 patients who conceived by ART also decided to terminate their pregnancies. Religious concerns are found to have a significant influence on decision-making process^18^.

The present study showed that psychological, economic, and religious factors were effective on the decision-making process. Findings from a study among the Muslim-Arab population also asserted that emotional, religious, and social factors had an effect on parents’ decisions^19^. Bryant et al. found that religion had a significant influence on patients’ attitudes toward termination^20^. A study by Bell et al.^21^ also found that decreased church attendance was correlated with a higher tendency to terminate the pregnancy.

Another previous study also found that psychological factors, concerns of career, and allocation of time and energy to families’ other children were effective on TOP decision^22^.

A review including 11 studies found that psychological factors significantly influenced the decision-making process following a prenatal diagnosis of trisomy 21. Psychosocial considerations—such as perceived parenting burden/reward, anticipated quality of life for a child with Down syndrome, attitudes toward and comfort with individuals with disabilities, and the level of social support emerged as important determinants^23^.

In this study, participants in the TOP group indicated that the information provided by medical professionals had a significant impact on their decision-making process. In contrast, the majority of the non-termination group reported that these counseling sessions did not substantially influence their decisions. Although in multivariate analysis the difference between the two groups was not found to be significant, this finding is noteworthy. As standardized sessions were conducted with both groups, ensuring equal time and attention was given to all parents. This suggests that individuals who decided to continue the pregnancy had largely made up their minds prior to the consultation, likely based on personal reasons independent of professional medical counseling.

A systematic review emphasizes the influence of healthcare professionals, particularly obstetricians and fetal medicine specialists, in the decision-making process. A counseling session in which up-to-date and accurate information is conveyed effectively and empathically to parents is considered essential^24^.

In the decision-making process, the central ethical principle is respecting the pregnant woman's autonomy. Studies emphasize the importance of non-directive counseling, where healthcare professionals provide comprehensive, unbiased information without steering the decision^25,26^. The bioethical perspective of decision-making after a trisomy 21 diagnosis highlights the need for respectful, individualized support that prioritizes informed consent, emotional well-being, and parental autonomy in a socially and culturally sensitive context.

Decision-making is often stressful and emotionally charged. Women and couples face grief, uncertainty, and societal pressure, underscoring the need for psychosocial support and sensitive, empathetic counseling. Some families choose to continue despite a trisomy 21 diagnosis, driven by moral, religious, or child-centered reasons. They value nonjudgmental professional support during their pregnancies and in the postpartum period.

The strength of the present study lies in its being conducted at a tertiary center with a high referral rate from across the country. This study explores the decision-making process from multiple perspectives to identify factors influencing patients’ decisions. However, a key limitation is its retrospective design. The survey was conducted with parents weeks or even months following their decision regarding pregnancy termination. So, there may be potential recall bias. Additionally, as a single-center study, the results may not be representative of all regions within the country.

## CONCLUSION

The present study researched the factors affecting parents’ decisions to terminate or continue the pregnancy in DS. In the literature, there are many studies aimed to examine the effective factors, but the results aren't compatible between these studies all the time. So multicentered studies with more participants may help to enlighten this decision-making process in DS.

## Data Availability

The datasets generated and/or analyzed during the current study are available from the corresponding author upon reasonable request.
